# Graph Mapping: A novel and simple test to validly assess fluid reasoning

**DOI:** 10.3758/s13428-022-01846-z

**Published:** 2022-04-19

**Authors:** Jan Jastrzębski, Michał Ociepka, Adam Chuderski

**Affiliations:** 1 Institute of Philosophy, Jagiellonian University, Grodzka 52, 31-044 Krakow, Poland; 2 Institute of Psychology, Jagiellonian University, Ingardena 6, 30-060 Krakow, Poland

**Keywords:** Reasoning, Fluid intelligence, Intelligence test, Gf, Working memory

## Abstract

We present Graph Mapping – a simple and effective computerized test of fluid intelligence (reasoning ability). The test requires structure mapping – a key component of the reasoning process. Participants are asked to map a pair of corresponding nodes across two mathematically isomorphic but visually different graphs. The test difficulty can be easily manipulated – the more complex structurally and dissimilar visually the graphs, the higher response error rate. Graph Mapping offers high flexibility in item generation, ranging from trivial to extremally difficult items, supporting progressive item sequences suitable for correlational studies. It also allows multiple item instances (clones) at a fixed difficulty level as well as full item randomization, both particularly suitable for within-subject experimental designs, longitudinal studies, and adaptive testing. The test has short administration times and is unfamiliar to participants, yielding practical advantages. Graph Mapping has excellent psychometric properties: Its convergent validity and reliability is comparable to the three leading traditional fluid reasoning tests. The convenient software allows a researcher to design the optimal test variant for a given study and sample. Graph Mapping can be downloaded from: https://osf.io/wh7zv/

An important facet of human cognitive aptitude (intelligence) is called fluid intelligence (Cattell, 1963), or fluid reasoning (Carroll, 1993). It is defined as “drawing inferences, concept formation, classification, generating and testing hypothesis, identifying relations, comprehending implications, problem solving, extrapolating, and transforming information” (McGrew, 2009, p. 5). Although fluid reasoning is only one of several broad abilities (including knowledge, quantitative reasoning, memory, etc.) contributing to general cognitive ability (the so-called *g* factor; Carroll, 1993), it is believed to most closely approximate *g* (e.g., Arendasy et al., 2008; Gustafsson, 1984). As a result, fluid reasoning tests have become a key screening tool in research on human cognition, such as in cognitive psychology, cognitive neuroscience, developmental psychology, and psychopathology, but have also shown high predictive value in applied contexts, such as academic achievement (Kuncel et al., 2001), career success (Schmidt & Hunter, 1998), and health (Deary, 2012). A growing line of research has investigated basic cognitive (e.g., Chuderski, 2014; Rey-Mermet et al., 2019; Unsworth et al., 2014) and neuronal factors (e.g., Barbey, 2018; Basten et al., 2015; Gągol et al., 2018) underlying performance on fluid reasoning tests, and has evaluated their (usually, very good) psychometric properties (e.g., Chuderski, 2013; Embretson, 1995; Gignac, 2015).

## Fluid reasoning testing

Fluid reasoning tests are typically comprised of nonverbal tasks that require inductive reasoning and spatial processing. Most of them involve the discovery of abstract rules governing relatively complex figural stimuli patterns and their application to find the response option that best completes these patterns. One widely applied test is Raven’s Advanced Progressive Matrices (RAPM; Raven et al., 1998), which presents the 3 × 3 matrices of geometric patterns, with a bottom right pattern missing. The test requires inducing this missing pattern from the structure of the row- and column-wise variation among the remaining patterns, including permutation, increase in number or value, and logical relations such as AND, OR, and XOR. Solution rates across consecutive RAPM items decrease from 90 to 10%, depending on several factors, such as the type and number of governing rules (Carpenter et al., 1990), as well as the number and complexity of figural stimuli (Primi, 2002). Similar matrix problems are part of another fluid reasoning test: Cattell’s Culture Free Intelligence Test (CFT-3; Cattell, 1961). Three other sections of CFT-3 require pattern series completion, shape categorization, and topological relation understanding. In another kind of fluid reasoning test – the four-term figural analogies (A:B::C:D; Snow et al., 1984) – the structure of geometric transformations must be identified between two patterns (A and B), and the same transformations must be applied to pattern C to infer the correct analogue of B (i.e., the correct option D which is to C as B is to A).

Although the high validity and reliability of the hallmark fluid reasoning tests have been supported by several decades of research, they seem to yield some methodological and practical problems, which, in the context of recently increasing interest in human intelligence understanding and testing, make these tests non-optimal measurement tools. The outburst of differential, developmental, cognitive, educational, vocational, and neuroscientific research involving cognitive ability, as well as increasing popularity of mobile technologies and online testing, seem to result in the substantial need for equally highly valid and reliable – but much more manipulative, interpretative and applicative – fluid reasoning tests, which would be devoid of the disadvantages of currently available tools.

First, the hallmark reasoning tests, including RAPM and CFT-3 (and their variants as well as other conceptually similar tests, e.g., BOMAT; Hossiep et al., 1999), are copyrighted, include a relatively small number of pre-existing items (e.g., 36 in RAPM), and can be administered in the paper-and-pencil format by qualified psychologists only. Although other tests (e.g., Ekstrom et al., 1976; Arendasy & Sommer, 2012) are openly available (hereafter called *open fluid reasoning tests*) as computer software allowing the generation of multiple items on demand (e.g., Sandia Matrices; Matzen et al., 2010), they are based on relatively simpler rules, and therefore might not fully tap into critical cognitive processes determining individual differences in fluid reasoning in the adult population. For instance, the Sandia matrices conceptually match Raven’s Standard Progressive Matrices, which is a variant of Raven’s test typically applied in child, aging, and patient samples (unlike RAPM). Even the most difficult of Sandia matrices – those based on logical operations – yield solution rates of around 40% in the adult sample (Matzen et al., 2010; see also Pahor, Stavropoulos, Jaeggi, & Seitz, 2019), whereas the late, most difficult items of the hallmark reasoning tests are solved around the random level (10–20% for items 30–36 in RAPM; Carpenter et al., 1990; Chuderski, 2013). Therefore, an open fluid reasoning test should be sufficiently manipulative to generate strongly demanding problems, in addition to easy and moderate problems, in order to capture the processes that are critical for the highest levels of fluid reasoning ability, allowing a representation of the entire ability range (similarly as is the case of the late items in such tests as RAPM, CFT-3, BOMAT, and so on).

Secondly, the design of most of the existing open fluid reasoning tests is relatively complex, meaning that multiple factors can potentially contribute to the scores. For instance, Sandia matrices, deriving its design from the original Raven test, requires abstracting the key geometric transformations from the perceptual input (which is not easily detectable in the case of overlaid figures), then discovering the rules governing these transformations, followed by constructing the missing element and/or inspecting the response options in search for a cue, and, finally, comparing the most plausible options to select the correct one. On the one hand, people differ not only in sheer reasoning ability, but also in visuospatial abilities (see Miyake et al., 2001) and strategies of coping with response options (adopting either a constructive or eliminative approach; see Jarosz et al., 2019). Therefore, it is highly difficult to interpret the resulting scores as indicating a single ability (see Gignac, 2015). On the other hand, important test requirements, for example rule discovery, might in fact be irrelevant for capturing fluid reasoning, as the test variants with the rules revealed to participants show psychometric properties comparable to original tests (e.g., Chuderski, 2013; Loesche et al., 2015). Therefore, an optimal open reasoning test should minimize all irrelevant requirements potentially uncovering its key requirement for fluid reasoning, and at the same time should involve as simple and interpretable component of a solution process as possible.

Finally, in the accelerating world and pervasively online communication, tests such as RAPM and their computerized variants involve impractical administration. Completion of each test item typically takes a minute or so (e.g., 40 min is allowed for 36 RAPM items within the standard procedure), whereas decreasing the number of items or time allowed per item always yields a risk of reliability/validity attenuation (Chuderski, 2013). Moreover, matrix problems, geometric analogies, and spatial tasks have already been popularized in the media, and thus they need not appear as “novel” and “abstract” for some participants who can benefit from their familiarity (see Schneider et al., 2020). Finally, the presence of response options can influence the reasoning processing in question, either by disturbing it so that incorrect options that are highly similar to the correct option can easily capture attention even if the reasoning process was potentially leading in the correct direction (see Jarosz & Wiley, 2012; Vodegel Matzen et al., 1994), or by facilitating it, where high dissimilarity between correct and incorrect options allows easy response elimination even if the problem itself is not easy (Arendasy & Sommer, 2013; Chuderski, 2015). Tests that require responses to be constructed because no response options are available may thus show increased validity as fluid reasoning measures (e.g., Becker et al., 2016). Therefore, an open fluid reasoning test, which would rely on a novel paradigm that is unfamiliar to the population and which allows valid testing within a short time and without involving response options, might potentially improve the available methodology of fluid reasoning measurement as compared to existing tests.

We propose that a benchmark test for assessing fluid reasoning ability, which captures crucial processes and mechanisms involved in effective fluid reasoning, consists of the mapping of two structures of relations. Structure mapping was originally proposed as the key component of analogical reasoning (Gentner, 1983). It is a process responsible for finding the most explanatory univocal structural correspondence between two situations by matching together those elements (objects, features, relations) that play the same relational roles across the two situations, irrespective of their semantic (i.e., non-relational) associations. For instance, when seeing a girl chasing a dog grabbing a toy as well as a mother shouting on a boy for stealing a dessert, one can correctly map the boy to the dog despite that they belong to two different categories (as well as map the toy to the dessert, chasing to shouting, and so on). Typically, one-to-one mappings are preferred (an argument to an argument, a relation to a relation). After establishing the valid correspondence between the relational structures describing the situations, one can also use knowledge of a more familiar situation to infer the structurally matching elements of a less familiar situation. Finding structural correspondence loads strongly on cognitive capacity, including working memory and attention control, and the requirement to map more than a few relational elements leads to frequent errors (e.g., Birney & Bowman, 2009; Cho, Holyoak, & Cannon, 2007; Halford, Baker, McCredden, & Bain, 2005; Veale & Keane, 1997). More recently, structure mapping was considered the key mechanism for a range of reasoning processes beyond analogy (Holyoak, 2012), including fluid reasoning (Lovett & Forbus, 2017). Moreover, conceptualizing fluid reasoning as relational structure mapping fits into recent theorizing on fluid reasoning ability as reflecting primarily the effectiveness of representing, processing, and integrating relations (Chuderski, 2019; Halford et al., 2014; McLoughlin et al., 2020; Oberauer et al., 2008).

Specifically, we have developed a novel test (*Graph Mapping*) that requires identifying two pairs of nodes that correspond structurally across two mathematically isomorphic but visually distinct graphs. Such a task instantiates structure mapping in a pure and abstract form, with graphs being a prototypic relational structure, presented in the simple visual form. Even a relatively difficult test item can be completed in about 20 s (although even more complex items are also possible), and requires neither rule discovery, as the task is explicit and can be pre-trained, nor option selection, as a response has to be generated as the sequence of two mouse clicks.

## The Graph Mapping test

**Fig. 1:**
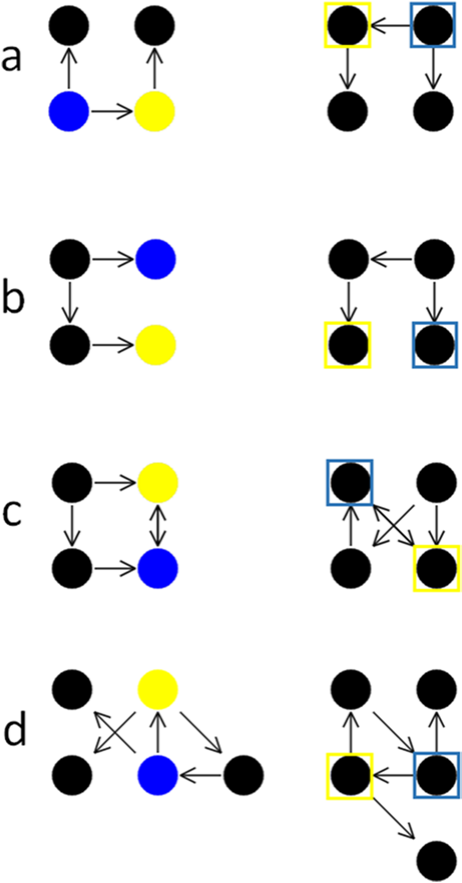
Four examples of the Graph Mapping trials. *Note*. Panels a, b, and c present examples of the Graph Mapping trials applied in Study 1: **a** The simplest direct type of targets, in which each target node can be identified by analyzing its incoming and outgoing edges. **b**, **c** More difficult, indirect type of targets, in which the structure of edges must be considered to some extent. **c** Includes the crossed edges condition, in which in one of the graphs the edges were crossed for additional difficulty. **d** An example of a relatively more complex test trial from Study 2. The *blue* and *yellow frames* indicate the correct responses (not shown in the real task)

In each trial of the Graph Mapping test, two relationally isomorphic directed graphs are presented on the screen (Fig. 1). The location of nodes in the right-hand graph is changed relative to the left-hand graph while preserving the same structure of connections (edges) between the nodes. The location of graphs on the screen can be set up flexibly, e.g., switched in cultures which read from the right to the left.

Each graph is displayed as a pattern of circles connected by uni- or bi-directional arrows. Two target nodes in the left-hand graph are highlighted; for example, one can be blue and the other can be yellow. The goal is to find and mark the two corresponding nodes in the right-hand graph by clicking on the selected nodes with the left and the right mouse button for the blue and the yellow node, respectively. A one-target variant of the task is also possible, but the two-target variant offers a lower guessing probability rate and thus a better reliability. The task allows to create a virtually unlimited number of unique graphs/trials ranging from trivial (such as a graph with two nodes connected by a single directed edge) to virtually unsolvable (e.g., complex graphs with up to 20 edges), making Graph Mapping highly suitable for use across the full range of cognitive ability.

### Presumed process of Graph Mapping

To successfully perform the mapping in a limited amount of time, a solver needs to efficiently recognize the abstract structure of the two graph instances regardless of their physical appearance. This requires identifying the specific nodes and discriminating between them in the mind. More specifically, we assume that the following processes are involved.

First, a given node can be directly identified by the specific number of its incoming and outgoing edges (the node’s degree). We assume that encoding a degree requires binding of the specific node with the number of its incoming edges and the number of outgoing edges. Such a relation may be binary when there is only one kind of edge (e.g., “the node has one incoming edge”), and the other one does not need to be represented, or may have two arguments (e.g., “one incoming and two outgoing edges”). Such a relational representation needs to be reliably maintained in the mind.

Next, to discriminate among the nodes and to establish their correspondence, the above node-degree relation needs to be compared to the relations with respective degrees for other nodes in order to validate whether the relations in question are the same or not. Discriminating between the nodes on the basis of a simple perceptual distinctiveness may work when comparing nodes with substantially distinct degrees or comparing a node with one connected edge with a node with two or three connected edges. However, it probably would not be effective when comparing nodes of similar degrees, or when comparing a node with one incoming edge with a node with one outgoing edge.

When the comparison of the nodes’ degrees does not suffice to map them across the two graph instances, or could in principle suffice but is difficult and can be additionally facilitated, then analyzing the connections between the nodes may be helpful in order to identify the specific role of a node in the graph’s structure. Representing such connections may require assigning the proper nodes to proper roles within a relatively complex relation (e.g., “this specific node connects to the node with two incoming edges”). In particular, discriminating between nodes of exactly the same degree can only be achieved by taking into account their connections with some or all of the other nodes (Fig. 1b).

The key requirement of the Graph Mapping task thus seems to consist of the need to maintain reliable relational bindings in the mind, while concurrently processing other pieces of relational information. Therefore, errors in the task likely result from misrepresenting the degrees of the nodes and/or the relations between the nodes. We assume that successfully establishing the graph structure requires identifying the nodes validly as well as discriminating correctly between them. That aim can be achieved threefold: when possible (in easier items), on the basis of the node perceptual distinctiveness (e.g., a single node with one connected edge stands out clearly from the nodes with two or three connected edges); when perceptual distinctness is low (in moderate items), by identifying and comparing their degrees explicitly; and when degrees are uninformative (in difficult items), by attending to a larger structure of relations between the nodes.

The difficulty of the mapping process should, consequently, be affected by such graph characteristics that (i) hinder the reliance on simple perceptual distinctiveness and thus force the solver to process the degrees explicitly; (ii) make processing of node degrees insufficient to map the graph and thus force the solver to attend to the entire structure of the graph; (iii) increase the graph’s complexity making (i) and (ii) even more demanding, in line with existing studies on the cognitive load imposed by relation processing (e.g., Birney & Bowman, 2009; Cho et al., 2007; Halford et al., 2005).

In two studies, Graph Mapping was validated as a reliable fluid reasoning test that can be manipulated more flexibly, better interpreted, and administered in an easier way, as compared to many established tests (e.g., matrix reasoning tests). The primary goal of the first study was to verify the above theoretically motivated factors potentially affecting the mapping process, i.e., the perceptual similarity of the two graph instances, the need to establish the graph structure, and the level of its complexity, the latter defined by the number of its nodes and corresponding edges. Manipulating these three factors should enable precise adjustment of the difficulty level of test trials, which firstly would be highly useful in experimental settings (e.g., testing the effects of varied difficulty on specific variables), and secondly would allow precise targeting of the test variants on various samples, from highly performing young adults to young children, elderly, and clinical groups. The main goal of the second study was to examine convergent validity of Graph Mapping by testing its relationship to the three above-mentioned hallmark tests of fluid reasoning (RAPM, CFT-3, and geometric analogies), as well as investigating its divergent validity, by observing its deviation from the working memory (WM) construct, which is the strongest known predictor of fluid intelligence (Oberauer et al., 2005). Both studies also verified the psychometric properties of the test in terms of the proper distribution of its scores, their good test–retest reliability (Study 1), and high internal consistency (Study 2). We also analyzed the minimum number and type of test trials that still allow measuring fluid intelligence effectively.

## Study 1: Factors affecting difficulty of the Graph Mapping test

In line with the research on relational reasoning (e.g., Bateman, Thompson, & Birney, 2019; Chuderski, 2014; Halford et al., 2005; Primi, 2002), we assumed that the cognitive demands imposed by the task should depend on the graphs’ perceptual and relational complexity. Such a complexity should naturally result from the size of the graph – the number of its nodes and edges – which affects the number of elements that need to be identified, navigated through, and mapped. The greater the number of edges, the larger the degrees need to be processed, which requires processing relations with larger numbers of arguments (compare Fig. 1d and a). Nodes with larger degrees are also more difficult to discriminate basing simply on their perceptual distinctiveness.

Second, the difficulty should depend on the extent to which the task can be solved solely by direct identification of nodes’ degrees (relatively easier; see Fig. 1a) versus the need to consider a larger structure of relations between the nodes (relatively more difficult; Fig. 1b). In other words, it matters whether the target nodes either needn’t or must be identified indirectly using an additional solving step in which the relations between the targets and the other nodes are considered.

Third, the process of mapping should be hindered by large perceptual distinctiveness of the graph instances. It should be easier to match the structure of graph instances of a similar “shape,” for example, when both instances do not contain the diagonal edges crossed (Fig. 1a), as compared to graph instances of strongly differing “shapes,” for example when one of the instances contains crossed edges (Fig. 1c). In the latter case, discrimination of nodes between the two graph instances basing solely on their perceptual distinctiveness is limited to a large extent, and thus their degrees must be compared explicitly.

The effect of these three easily manipulable factors on performance accuracy was examined in the following experiment. First, the *number of edges* (three, four, and five) was varied across the graphs. Second, different *types of targets* were applied. In the easiest type, both target nodes could be identified directly by the unique degree of each target (Fig. 1a). In contrast, in the most difficult type, the targets could not be identified directly, as they both had the same degree; therefore, other nodes needed to be identified directly first, and then the targets could be identified by their unique connection with the former nodes (Fig. 1b). In the mixed case, one target was of direct and another of indirect type. Third, in a random half of trials, one random graph instance had two diagonal *edges crossed* (Fig. 1c), while in the other half, both graph instances had all the edges uncrossed, facilitating their mapping. Participants completed the task twice. By correlating performance between the two sessions, the test–retest reliability of Graph Mapping could be assessed.

### Participants

The sample size of around 60 participants was sufficient to achieve very high power for small effect size in the within-subjects design. Sixty-three participants (37 women) were recruited using ads on popular websites and were paid the equivalent of 10 euros for participation. Their mean age was 21.7 years (SD = 2.7, range 18–30). The participants were informed that participation was anonymous and could be ended at will at any moment. Two participants were excluded because of their very low response times in a substantial number of trials and very low solution rates, reflecting insufficient engagement. The final sample included 61 participants of which 56 completed both sessions.

### Procedure

The study took place in a psychology laboratory at a central European university. Participants were tested individually. They completed Graph Mapping twice; the second session took place 1 week after the first session. Each session consisted of three blocks of trials applied consecutively. Each block consisted of 18 trials – one trial for each factor levels combination, presented in a random order. One session included 54 experimental trials in total. They were preceded by instructions that included two examples with correct responses explained, and by a practice session with 12 trials with feedback. To ensure that participants properly understood the task, the experimental trials started only if the accuracy on the practice trials reached the pre-specified satisfactory level (70%). Participants had a maximum of five practice attempts to learn the task, but most of the participants needed just one attempt. The trial time limit equaled 24 s. Participants were advised that only accuracy on the tasks counted, not speed. A small clock icon was shown 5 s before the trial time limit to notify the participant of the brief time remaining. A half-minute break was applied in the middle of the test. A small mouse icon with the left and right button marked in the respective colors of the two targets was always shown as a reminder at the bottom of the screen.

Each graph in the task had four nodes forming a square shape. Importantly, across all the trials, all the graphs within the same number of edges were generated from a single template graph (they were mutually isomorphic). This enabled control over the influence of any potential confounding variables that could be related to the specificities of different graphs’ structures and to validly compare the consecutive number-of-edges conditions (i.e., more edges would allow for more distinct graphs, if not controlled for). To introduce randomization, in each trial the exact visual form of each graph instance was random, meaning that the placement of its nodes was randomized given the constraints (i.e., whether the edges can cross or not). Equally probable transformations of each template graph included rotations by 0/90/180/270 degrees and mirror reflections across the *X*- and *Y*-axis as well as two diagonals of the template graph. Rotation and mirror reflection preserve the “shape” of the graph as they neither introduce nor remove any diagonal edges, and thus the number of edges crossed (present or absent in the template graph, depending on the condition) remains constant. In sum, each instance of a graph in a trial was a random variant out of eight possible variants (four rotations and four mirror reflections) of the template graph. Thus, each trial consisted of a random pair of graph instances selected out of 64 (8 x 8) possible pair variants. Correctly matching each of the two targets (blue and yellow) across the two graphs (in any order; blue before yellow or vice versa) counted as the correct response. The individual score on the task equaled the number of correct responses out of all 54 trials.

### Results

The distribution of scores well approximated the normal distribution, as indicated by skew (0.24) and kurtosis (– 0.83) (criterion values |2.0| and |4.0|, respectively). The mean score in both sessions equaled M = .57 (SD = .16, range .27 to .91). The results of ANOVA, with the three task conditions as factors (the number of edges: 3, 4, or 5; edges either uncrossed or crossed; direct, mixed, or indirect identification of targets), and with mean accuracy as a dependent variable, are shown in Table 1. Each factor had a highly significant effect on accuracy in the direction predicted. However, it appeared that the mixed targets condition provided relatively inconclusive results, sometimes yielding a performance similar to direct targets, and in other cases the one resembling indirect targets. Therefore, this condition was only included in ANOVA, but for clarity was not presented on the following figures (the figures including the mixed condition can be found at https://osf.io/wh7zv/).

**Table 1:** The results of ANOVA in Study 1 showing the effects of three manipulated conditions and their interactions

Factors	Sum of squares	*df*	Mean square	F	*p*	η^2^_p_
No. of edges	92.443	2	46.221	218.749	< .001	0.065
Crossed	74.702	1	74.702	353.538	< .001	0.053
Targets	26.827	2	13.414	63.482	< .001	0.020
No. of edges ✻ Crossed	1.272	2	0.636	3.009	0.049	< 0.001
No. of edges ✻ Targets	10.112	4	2.528	11.964	< .001	0.008
Crossed ✻ Targets	3.217	2	1.609	7.614	< .001	0.002
No. of edges ✻ Crossed ✻ Targets	9.653	4	2.413	11.421	< .001	0.007
Residuals	1331.185	6300	0.211			

**Fig. 2:**
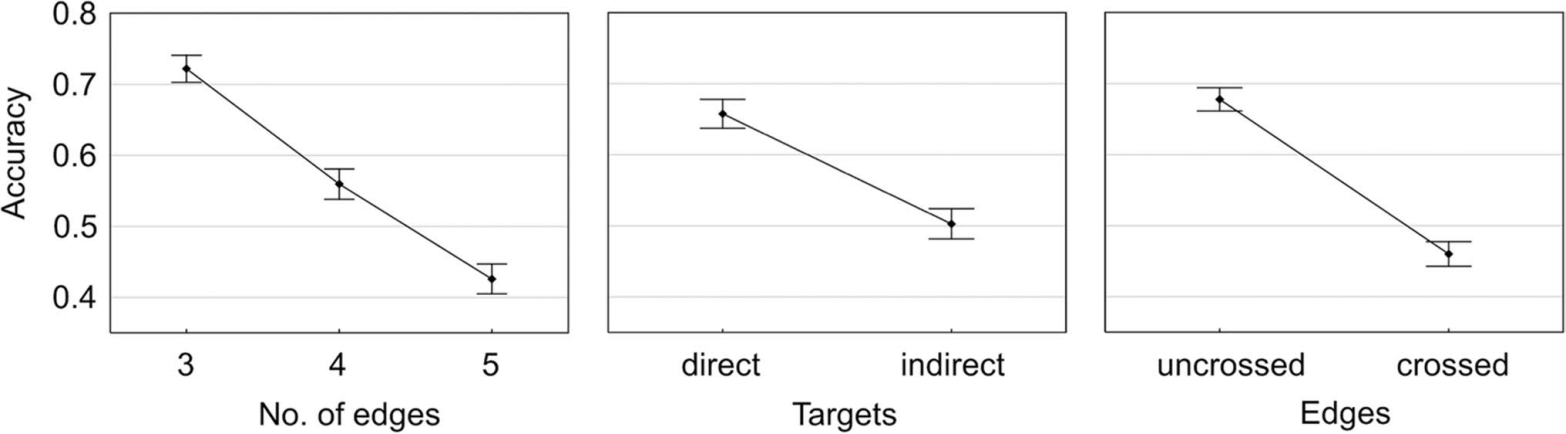
The main effect on accuracy rates of each of the three test conditions manipulated in study. *Note*. *Vertical bars* represent 95% CIs

**Fig. 3:**
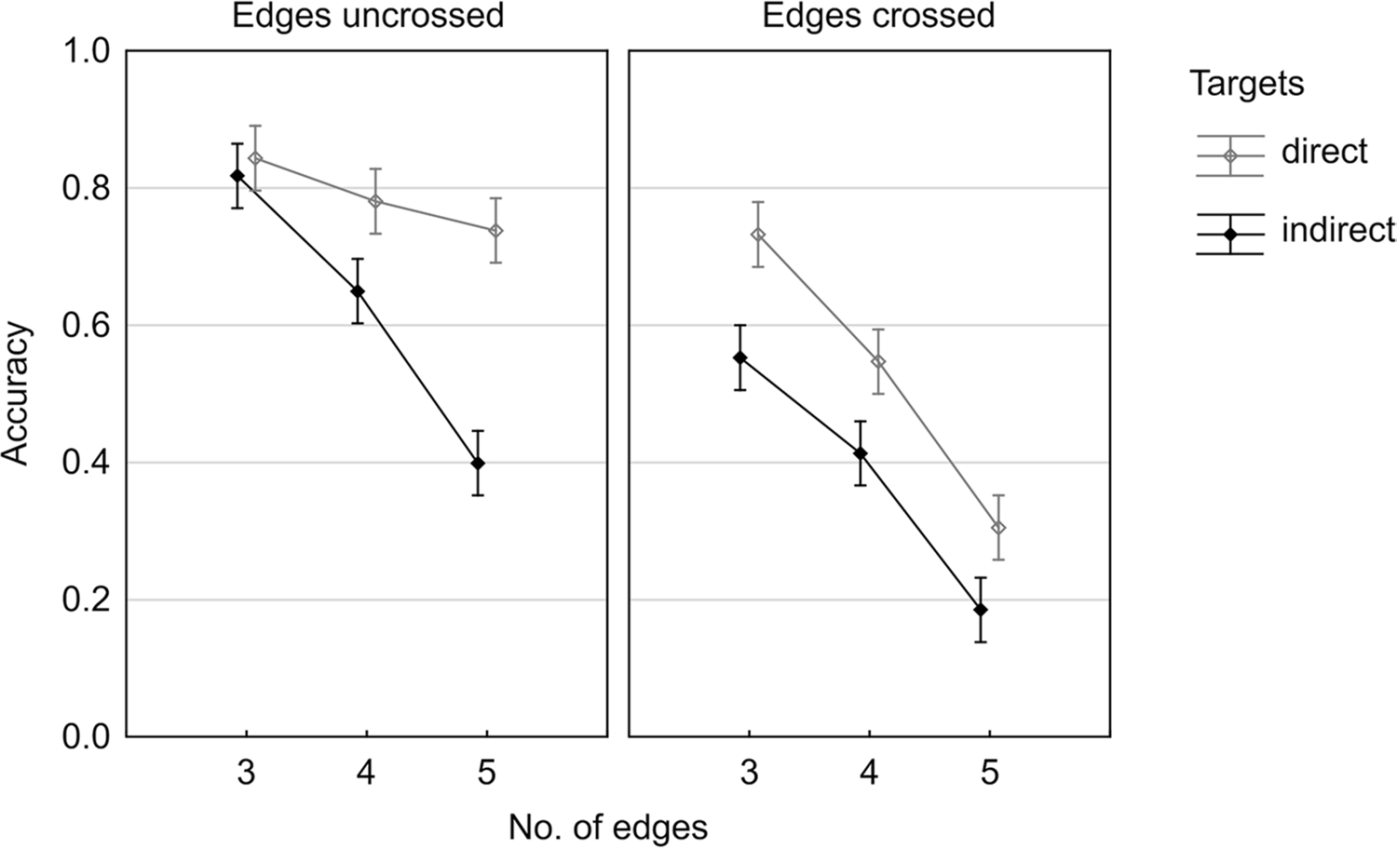
The effects on accuracy rates of the three test conditions manipulated in Study 1. *Note*. *Vertical bars* represent 95% CIs

Figure 2 presents the accuracy rates across the test conditions. As predicted, the direct targets were much easier to process, as compared to the indirect targets, with the only exception for the easiest condition of uncrossed three-edge graphs, but this lack of effect was due to ceiling in this condition. The accuracy in particular trials (Fig. 3) ranged from M = .84 for the three-edge, non-crossed, direct targets graphs to M = .19 for the five-edge, crossed, indirect targets graphs (random level was .083). The results show that the task difficulty can be predictably controlled for by manipulating the three factors adopted. There was a three-way interaction of all the factors, indicating that the effect of the number of edges became amplified if only one of the remaining factors was present (either indirect targets or edges crossed, or both), as compared to their easiest combination (direct targets and edges uncrossed).

The test–retest reliability of Graph Mapping was very good, as shown by the strong correlation between the scores on two sessions, *r* = .76**,** 95% CI = [.63, .86], showing that the task is a reliable indicator of stable individual differences. For comparison, the test–retest reliability of full RAPM, with 40 min of administration time, is around .85 (Bors & Forrin, 1995), but drops to around .75 for the shortened RAPM version (Arthur & Day, 1994; Arthur et al., 1999), which had a comparable administration time to Graph Mapping, ranging around 15 min.

**Fig. 4:**
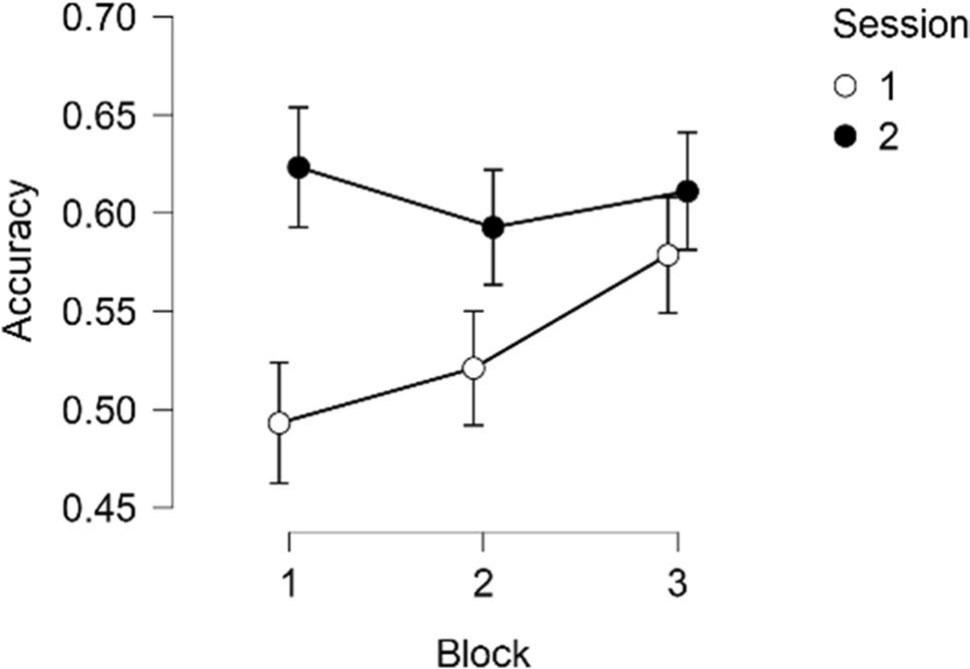
**M**ean accuracy on three consecutive blocks of two subsequent test sessions in Study 1. *Note*. Vertical bars represent 95% CIs

Figure 4 shows the learning effect taking place between blocks of the first session. In the second session, the performance stabilized across the blocks, and was close to the performance on the last block of the first session. Thus, unsurprisingly, there was some effect of practice on the early trials of the test. However, performance reached an asymptote after the first 36 trials, suggesting some fundamental limits that could not be eliminated via learning and transfer.

## Study 2: Convergent and divergent validity of the Graph Mapping test

In the second study, Graph Mapping was administered in another potentially useful form, more similar to a typical reasoning test. All participants completed the same sequence of unique predefined trials (henceforth called items, to distinguish them from automatically generated trials), applied in a fixed, progressive difficulty order. The validation of the test’s psychometric properties was conducted as part of a larger experiment designed to evaluate the hypothesis claiming equivalence between the ability to effectively validate simple relations – measured, among others, by Graph Mapping – and fluid intelligence, measured by three leading fluid reasoning tests. That experiment also examined whether the former ability can be distinguished from WM, measured by three typical WM tasks. The theoretical goal of the present analysis was to determine the validity of Graph Mapping as an effective fluid intelligence test (its convergent validity) and not just another WM task (divergent validity). For a full description of the procedure, other measures applied in the study, and data handling, as well as a full description of the results, please see Jastrzębski et al. (2020). Here, we present only the results of the validation of the Graph Mapping task.

### Participants

The recommended rule of thumb for the sample size in a SEM study is at least 200 participants (Kline, 1998). Two hundred forty-five participants (154 women) were recruited using ads on popular websites; participants were paid the equivalent of 10 euros for their participation. Their mean age was 22.8 years (SD = 3.1, range 18–35). About half of the participants were students (56%). Sixteen participants (6.5%) were excluded from all the following analyses because of lack of proper engagement, as indicated by very low scores and very short response times. The final dataset consisted of 229 participants.

### Procedure

The Graph Mapping administration consisted of 44 unique items presented in the fixed order according to progressive difficulty. Most of the items were of the direct and mixed type; the number of edges varied between three and six; the number of nodes in each graph increased from three in relatively easier items up to six in more difficult items; and in some of the graphs, nodes were organized into shapes different than a simple square. Thus, the diversity of graphs/items was much greater than in Study 1 in order to maximize the score variance yielded by the task. The remaining features of the test were the same as in Study 1. The test score was the mean accuracy across all 44 items.

The three classical fluid reasoning tests, which were mentioned in the Introduction, were applied: Raven’s Advanced Progressive Matrices (RAPM; Raven et al., 1998) consisting of 36 problems, Cattell’s Culture Fair Intelligence Test (CFT-3; Cattell, 1961) composed of three subtests (Series, Classifications, Topology) including 36 problems in total (the Matrices subtest overlapped conceptually with RAPM, so it was skipped), and the Figural Analogies Test (Analogies; Chuderski & Nęcka, 2012) consisting of 36 analogies. Each test and subtest was divided into two equipotent sets of items. Each set was administered to a random half of participants in the typical paper-and-pencil format and to the other half in the computerized format. Such a division allowed comparing the Graph Mapping correlations between the paper-and-pencil administration and computerized administration of the fluid intelligence tests in order to exclude any role of the computer format of Graph Mapping on its capturing of the fluid intelligence construct.

Three WM tasks were applied: the spatial simple span task modeled after a respective complex span task (Conway et al., 2005), the figural complex span task (also modeled after Conway et al., 2005), and a variant of the mental counters task (Larson, Merritt, & Williams, 1988).

### Results

**Fig. 5:**
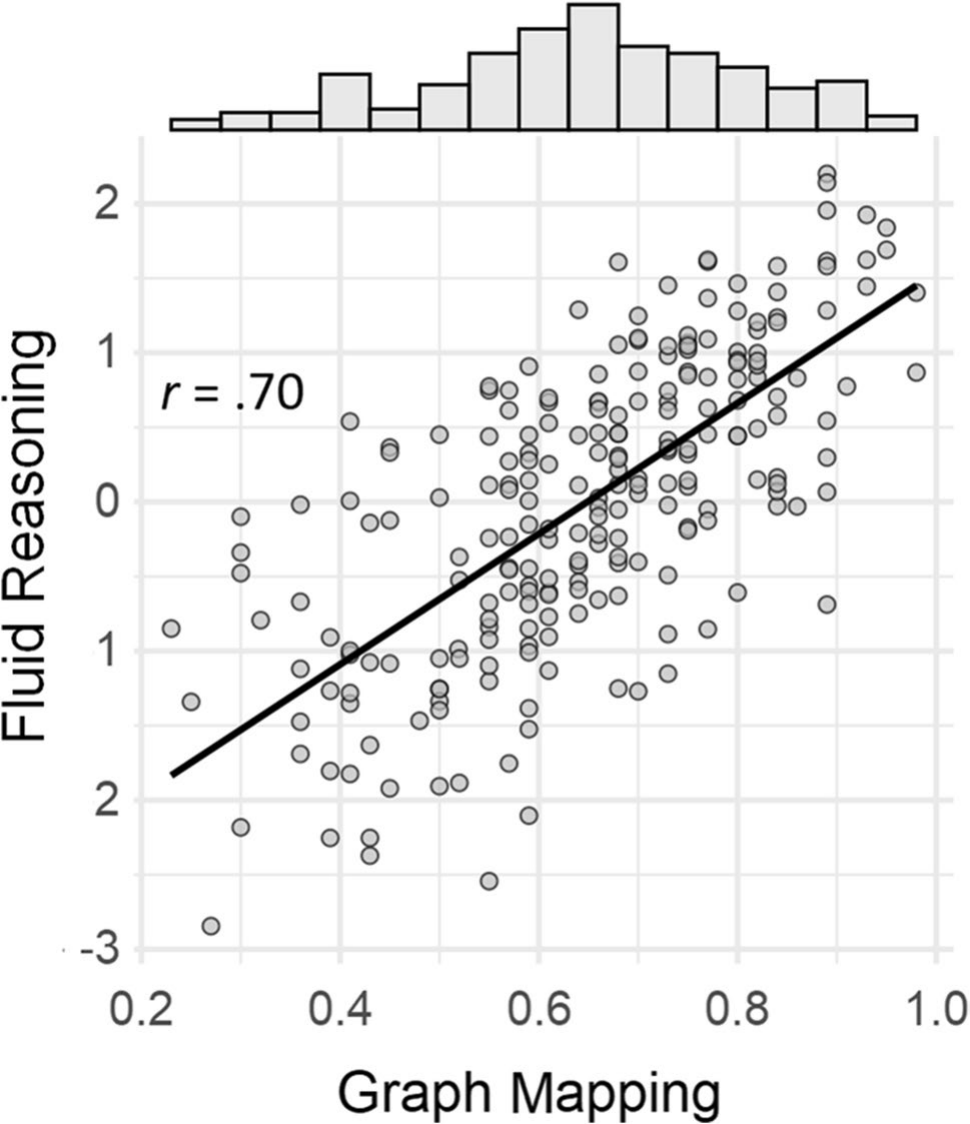
The relationship between accuracy on Graph Mapping and fluid reasoning factor (Gf). *Note.* The z-score on fluid reasoning factor was calculated from the three fluid reasoning tests. The histogram above the scatterplot shows the distribution of the Graph Mapping scores

The mean score on Graph Mapping equaled M = .65 (SD = .16, range .23 to .98). The scores’ distribution well approximated the normal distribution (Fig. 5), as indicated by low values of kurtosis (– 0.34) and skew (– 0.31). The internal consistency of the test, as indicated by Cronbach’s α = 86, was very good. The reliabilities of the reasoning and the working memory tests were at least satisfactory; α = .86, .75, and 72 for RAPM, Analogies and CFT-3, respectively; α = .74, .84, and .92 for Simple span, Complex span, and Mental counters.

The correlations (Table 2) between Graph Mapping and the three reasoning tests were visibly stronger (mean *r* = .61) than those between the former test and the WM tasks (mean *r* = .41). Figure 5 shows the correlation between Graph Mapping and the Gf factor, the latter calculated using scores on the three reasoning tests (principal component analysis, 76% variance explained, factor loadings λ = .90, .86, and .85, respectively). Also, for comparison, the correlations were computed between each fluid reasoning test and the variants of the Gf factor composed of the Graph Mapping test and the two remaining fluid reasoning tests. The resulting correlations equaled *r* = .75, *r* = .72, and *r* = .71, for RAPM, Analogies, and CFT-3. Therefore, the Graph Mapping correlation with the Gf factor was not significantly different from the respective correlations of the established fluid intelligence tests, each *p* > .083. Moreover, the Graph Mapping correlation with the Gf factor did not depend on the fluid reasoning tests format, as the correlations based on the computer and the paper format were almost identical (*r* = .66 and .64, respectively).

**Table 2:** Correlations between Graph Mapping, three fluid reasoning tests (RAPM, analogies, CFT-3), and three working memory tasks applied in Study 2

Measure		1.	2.	3.	4.	5.	6.
1.	Graph Mapping	-					
2.	RAPM	.60	-				
3.	Analogies	.61	.68	-			
4.	CFT-3	.61	.66	.58	-		
5.	Simple span	.51	.53	.41	.43	-	
6.	Complex span	.33	.38	.37	.34	.36	-
7.	Mental counters	.39	.41	.35	.35	.35	.29

**Fig. 6:**
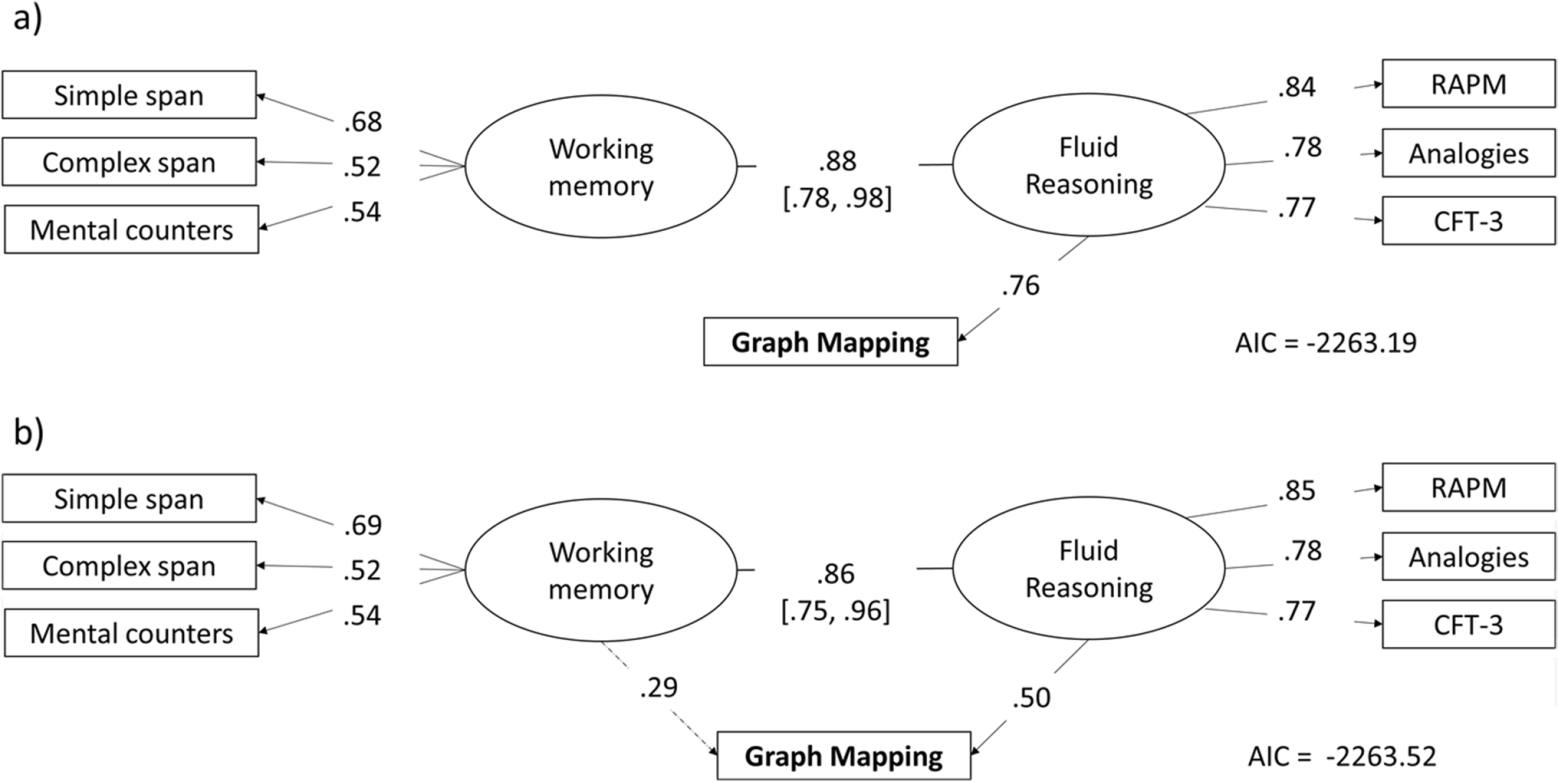
Two CFA models showing Graph Mapping loadings to fluid reasoning and working memory factors. *Note.* **a** Graph Mapping loads fluid reasoning factor only. **b** Graph Mapping is free to load both factors. The *ovals* represent factors and the *rectangles* represent manifest variables. The *values on the arrows* are standardized factor loadings, and the *values between the ovals* are correlation coefficients with 95% confidence intervals. The *dotted arrow* represents a non-significant loading. The values of Akaike’s information criterion (AIC) show that the fit of the two models is statistically non-distinguishable

The pattern of correlations was summarized using confirmatory factor analysis (CFA). In the first CFA model (Fig. 6a), Graph Mapping and the three reasoning tests all loaded on the Gf factor, the WM factor was loaded by the three WM tasks, while Graph Mapping loading on the WM factor was set to zero. The resulting loading of Graph Mapping on Gf was high (λ = .75) and comparable to the loadings of the reasoning tests (λ = .84, .79, 77, for RAPM, Analogies, and CFT-3, respectively). For comparison, when RAPM was free to load on both the WM and the Gf factor, while Graph Mapping, Analogies, and CFT-3 loaded only on the Gf factor, the RAPM-Gf loading dropped to λ = .69 while RAPM-WM loading was λ = .17 (non-significant).

In the second model (Fig. 6b), Graph Mapping was free to load on both WM and Gf factor simultaneously. Here, its Gf loading dropped to λ = .50, but was still significant, *p* = .004, while its WM loading was only λ = .29 and non-significant, *p* = .12. The difference in fit between the first, χ2(13) = 18.92, AIC = – 2263.19, and the second model, χ2(12) = 16.59, AIC = – 2263.53, was not significant, χ2 (1) = 2.34, *p* = 0.13, AIC difference = 0.34. Finally, the third model was tested in which Graph Mapping loaded WM but not Gf. The third model fitted the data worse, χ2(13) =19.94, AIC = – 2262.17, than both the first and the second model, though again, each difference was not significant, χ2 (1) = 3.36, *p* = 0.67, AIC difference = 1.36, for the difference between the second and the third model. However, these non-conclusive results should not be surprising, considering the extremally high overlap between WM and Gf factors, which shared as much as about 75% of variance, suggesting that *de facto* WM tests are also to a large degree accurate Gf tests, and vice versa. For this reason, formally demonstrating the full divergent validity of Graph Mapping was particularly challenging and would probably require a larger study sample.

Finally, in order to establish how much concise Graph Mapping administration still captured fluid reasoning validly and reliably, which seemed important from a practical point of view, the item samples of 22 even items and 22 odd items, as well as the samples of 15, 12, 11, and ten items (including every second, third, fourth item, etc.) were correlated with the Gf factor. The resulting correlations were compared to the full 44-item test correlation with Gf. The correlations equaled *r* = .64 to .67 for the two halves of the test. For the smaller samples, *r* oscillated around .6. Lastly, the correlation of the Gf factor with Graph Mapping, after removing its ten most difficult items, equaled *r* = .68, showing that the predictive power of the test did not rely on the most demanding items. We should notice that the above analyses should be treated with caution, as optimally they would require collecting data on independent, shortened versions of the test – as the selected samples were embedded in the full test, the learning and interference effects could influence their predictability. Overall, however, these results indicate that its useful predictive power might be achievable with as few as a dozen items (a variant administrable in less than 5 min) and proved the good internal consistency of Graph Mapping.

## Conclusions

We showed that the validity and reliability of the newly proposed computerized fluid reasoning test – Graph Mapping – is comparable to the three leading classical fluid reasoning tests; that is, the way Graph Mapping measures fluid intelligence is not inferior in any way, as compared to each of these tests. At the same time, Graph Mapping yields substantial advantages over these tests in several dimensions of test administration and interpretation.

One advantage is that, unlike matrix reasoning (comprising RAPM, BOMAT, Sandia, and one of the subsets of CFT-3), which is a complex task explained by multiple but often mutually contradictory theoretical models (e.g., Carpenter et al., 1990; Kunda et al., 2013; Little, Lewandowsky, & Griffiths, 2012; Rasmussen & Eliasmith, 2014), structure mapping has been investigated intensively for the last 40 years, and its theoretical models seem to converge into a well-defined, precisely constrained, incremental process of finding structural correspondence (e.g., Goldstone, 1994; Hummel & Holyoak, 2003; Keane, Ledgeway, & Duff, 1994; Larkey & Love, 2003; Lovett, Gentner, Forbus, & Sagi, 2009; but see Eliasmith & Taggard, 2001; Jani & Levine, 2000). Additionally, the attempts to identify the underlying cognitive process of structure mapping can be disturbed to a lesser extent, as compared to the established matrix reasoning tests, by the influence of potential strategies relying on some uncontrolled features of response options (see Arendasy & Sommer, 2013; Becker et al., 2016; Jarosz et al., 2019), because Graph Mapping simply includes no predefined response options; the response needs to be created. Therefore, in terms of theoretical validity, the processing underlying Graph Mapping can be relatively well understood and devoid of most of the task requirements, which are not essential for capturing fluid reasoning ability.

Second, the difficulty of particular items of classical matrix reasoning tests can be flexibly manipulated only in their simple variants, in which even the relatively demanding items are solved by almost half of the sample (Matzen et al., 2010; Pahor et al., 2019). In conceptually more complex variants, such as the original RAPM or the tests modelled after it, multiple factors affect the observed difficulty, and it is not possible to manipulate flexibly the difficulty level (e.g., Jarosz & Wiley, 2012; Primi, 2002). Graph Mapping allows difficulty variation ranging from ceiling to floor by means of only three precisely defined parameters: perceptual similarity, representational holism, and structural complexity. In fact, it allows for generating virtually unsolvable items within reasonable time limits, for instance, indirect nine-node items. Therefore, the task can be easily customized to any experimental purpose and any sample characteristic.

Third, Graph Mapping offers several practical advantages regarding test administration. The effective time necessary to complete the test is considerably reduced, as compared to 30–40 min required for completing RAPM and CFT-3. Specifically, 10–15 min allows for running the full-length procedure (e.g., 36 items), while less than 5 min suffices to administer a shortened but still highly valid and reliable procedure (e.g., 12 items). This flexibility to generate various variants of the test is another merit of Graph Mapping. In particular, the procedure allows a handy generation of either the fixed-order or randomized-order test variants. The former variants will be more suitable in correlational studies, granting that all participants receive the same form of test, as in typical individual differences research. The latter variants enable controlled experimental manipulations, particularly within-subject designs. There is also a virtually unlimited number of parallel variants possible – a valuable feature in longitudinal studies such as cognitive training. This latter feature combined with precisely controlled test item difficulty allows for the application of the adaptive procedures and the like. The highly convenient software provides every researcher with the possibility to design and run the optimal Graph Mapping variant for a given study.

Finally, the task to map two isomorphic but visually distinct graphs seems to be completely new for a substantial majority of potential solvers, and thus immune from any effects of familiarity and popularity. Relatedly, apart from some early trials (blocks 1 and 2), the task was not easily trainable (across blocks 3 to 6), so even some familiarity of the task, which is unavoidable in longitudinal designs, would most likely only minimally affect its validity; it would still reflect the fundamental cognitive limitation of an individual, and not the effects of acquired strategies. Moreover, as Graph Mapping does not resemble a typical intelligence test but looks as if it were a puzzle or a game, the solvers may not be able to recognize its actual purpose of measuring the individual fluid intelligence level.

In conclusion, we have proposed a novel method of fluid reasoning assessment, which yielded score range, reliability, and validity comparable to established complex fluid reasoning tests, while being highly simplified and relying on a relatively clearly interpretable cognitive process: structure mapping. The task difficulty is fully manipulative from ceiling to floor, and the requirements underlying easy versus hard items seem to be precisely defined. In addition to these methodological merits, the novel test offers several practical benefits: high flexibility in item generation, short administration, and test novelty. We believe that Graph Mapping, by enhancing the measurement of fluid reasoning performance and improving the understanding of processes underlying it, can help advance research involving reasoning and intelligence screening in multiple basic, clinical, and applied contexts, across various subdisciplines of psychology, education, management, and neuroscience.

## Downloading and running Graph Mapping

Graph Mapping runs on Windows and Linux operating systems. It was implemented using the Python 3 programming language and PsychoPy package (both free and easy to install, which is required for the task to work). The test is freely available and can be downloaded from: https://osf.io/wh7zv/

The test can be easily customized by using a configuration panel. Most importantly, it can be applied in two different modes. In the first mode, a researcher may want to design a custom randomized experiment, controlling the complexity/difficulty of the trials by selecting specific levels of each of the three features of the graph, as validated in Study 1: the number of edges (from 3 to 5), the presence or absence of crossed edges in one of the graph instances, and the type of targets (either direct or indirect). By default, each graph in each trial is randomly transformed visually, providing the uniqueness of each run of the experiment, while allowing for control over the key factors affecting the task difficulty.

In the second mode, a researcher may want to apply one of the ready-made tests from the set of tests of different length and difficulty level, including the default test based on the validated 44 items applied in Study 2. Each test includes a number of unique items presented by default in a progressive difficulty order, similarly as in a typical intelligence test. Optionally, it is also possible to set the random visual transformations of graphs – similarly to the first mode – as well as the random order of trials in order to create a unique test for each run of the task, while keeping the difficulty level of each run constant. Alternatively, instead of applying the ready-made tests, it is possible to modify the existing tests or create new custom graphs/trials and tests from scratch. It is then possible to apply such a new test in the same way as the ready-made tests.

The configuration panel enables other options as well, including customizing the training session and adjusting other settings such as various temporal and visual parameters of the procedure. The electronic manual provided with the Graph Mapping test explains its use in detail.

## Data Availability

The datasets are available in the OSF repository: https://osf.io/wh7zv/

## Code availability

The software is available in the OSF repository: https://osf.io/wh7zv/
